# Low back pain and osteoarthritis pain: a perspective of estrogen

**DOI:** 10.1038/s41413-023-00280-x

**Published:** 2023-08-04

**Authors:** Huiwen Pang, Shihui Chen, David M. Klyne, David Harrich, Wenyuan Ding, Sidong Yang, Felicity Y. Han

**Affiliations:** 1 Australian Institute for Bioengineering and Nanotechnology, The University of Queensland, St. Lucia, Brisbane, QLD 4072 Australia; 2 NHMRC Centre of Clinical Research Excellence in Spinal Pain, Injury and Health, The University of Queensland, St. Lucia, Brisbane, QLD 4072 Australia; 3 Department of Cell and Molecular Biology, QIMR Berghofer Medical Research Institute, Herston, QLD 4006 Australia; 4 Department of Spine Surgery, The Third Hospital of Hebei Medical University, 139 Ziqiang Road, Shijiazhuang, 050051 China; 5 Hebei Joint International Research Center for Spinal Diseases, 139 Ziqiang Road, Shijiazhuang, 050051 China

**Keywords:** Bone, Pathogenesis

## Abstract

Low back pain (LBP) is the world’s leading cause of disability and is increasing in prevalence more rapidly than any other pain condition. Intervertebral disc (IVD) degeneration and facet joint osteoarthritis (FJOA) are two common causes of LBP, and both occur more frequently in elderly women than in other populations. Moreover, osteoarthritis (OA) and OA pain, regardless of the joint, are experienced by up to twice as many women as men, and this difference is amplified during menopause. Changes in estrogen may be an important contributor to these pain states. Receptors for estrogen have been found within IVD tissue and nearby joints, highlighting the potential roles of estrogen within and surrounding the IVDs and joints. In addition, estrogen supplementation has been shown to be effective at ameliorating IVD degeneration and OA progression, indicating its potential use as a therapeutic agent for people with LBP and OA pain. This review comprehensively examines the relationship between estrogen and these pain conditions by summarizing recent preclinical and clinical findings. The potential molecular mechanisms by which estrogen may relieve LBP associated with IVD degeneration and FJOA and OA pain are discussed.

## Introduction

Low back pain (LBP) and osteoarthritis (OA) are two of the most common, disabling, and costly health conditions.^1^ LBP affects up to 80% of people at some point in their lives, with the majority of episodes occurring at the lumbar or waist region of the spine, which bears the brunt of the weight of the upper body and explains why LBP is so common in humans compared to quadruped animals.^2,3^ Many studies highlight intervertebral disc (IVD) degeneration and facet joint osteoarthritis (FJOA) as two major contributors to LBP.^4–6^ OA, on the other hand, is a progressive degenerative disease that tends to worsen over time and, as a result, frequently leads to chronic pain. OA pain can affect various joints in the body, including the hips, knees, hands, and spine. Both of these pain conditions place enormous health and economic burdens on patients, their families, and society.^7^

LBP prevalence and incidence rates are higher in women than men.^8,9^ Moreover, middle-aged women after menopause are more likely to suffer from LBP than their younger counterparts. Similarly, women are more likely to suffer from OA than men, with the prevalence increasing after menopause^10^. Hormonal changes that occur in older women as estrogen production slows may have a role.^11^ In premenopausal women, estradiol levels are typically between 30 and 300 pg·mL^−1^, whereas in postmenopausal women, levels typically fall to between 0 and 40 pg·mL^−1^.^12^ Estrogen deficiency, which occurs in more than 50% of perimenopausal (just prior to menopause) women, is closely associated with decreased bone mineral density and increased musculoskeletal pain.^13^ Women with severe menopausal symptoms are more likely to suffer from chronic back pain and OA.^10,14^ Moreover, estrogen receptors have been identified within the IVDs and different joints.

These links between estrogen and musculoskeletal pain contribute to the growing argument that estrogen is much more than just a sex hormone. In this review, we provide an overview of the relationship between estrogen and these pain conditions by summarizing current preclinical and clinical findings. The potential molecular mechanisms by which estrogen may relieve LBP associated with IVD degeneration and FJOA and OA pain are discussed. New avenues of research are presented.

### Intervertebral disc (IVD) degeneration

Like all musculoskeletal pain, LBP involves contributions from various biopsychosocial and lifestyle factors, especially as the pain continues to persist.^15,16^ One of the primary triggers, however, is IVD degeneration.^6^ IVD degeneration refers to a deterioration of the discs between vertebral bodies (hence the term “intervertebral”) of the spine, which can lead to discomfort, reduced range of motion, and various other symptoms associated with LBP.^17,18^ There are 23 IVDs in the human spine that lie between adjacent vertebrae, providing both the required support and elasticity for the spine.^19^ The IVD consists of three main components: the nucleus pulposus, the annulus fibrosus, and the cartilage endplate.^20^ IVDs function as vital fluid pumps, pushing water into the medullary canaliculi when under pressure and regaining water through the hydrophilic activity of proteoglycans when pressure is released.^21^ The viscoelasticity of IVDs decreases with age due to age-related reductions in the hydrophilic proteoglycan content and quality.^22^ IVD degeneration is characterized by reduced synthesis of proteoglycans, decreased water content, abnormal extracellular matrix (ECM) production, and improper collagen types.^23^ As the discs continue to lose elasticity and water content, they also lose their ability to resist compression and torque and maintain structural integrity. Compounding the problem is a cascade of consequential effects, including altered spinal biomechanics, neovascularization, and neoinnervation. These degenerative changes can alter the rest of the spinal column and adversely affect other spinal structures, such as muscles and ligaments, leading to the degeneration of adjacent tissues, nerve impingement (and radicular pain) and spinal stenosis, among other contributors to pain.^24^ These effects are amplified in older adults, as cells within IVDs and nearby tissues are more exposed to conditions (such as oxidative stress and damaged matrix products) that enhance cell death and senescence.^25–27^ Eventually, a pathologic state may be reached, and chronic pain occurs.^23^

### Facet joint osteoarthritis (FJOA)

FJOA is a consequence of spinal degeneration and is considered another primary cause of LBP. FJOA is a whole-joint failure disease that results from injury to one or more components of the spinal motion segment.^28–31^ In humans, facet joints are a set of synovial joints located between the articular processes of two adjacent spinal levels. The facet joints and IVD, also known as the ‘three-joint complex’, constitute an important component of spinal motion segments.^32^ The ‘three-joint complex’ is crucial for enabling spinal motions, including rotation, flexion and extension.^33^

### Osteoarthritis (OA)

OA is a degenerative joint disease, a common musculoskeletal disorder in elderly people, that is characterized by the deterioration of cartilage, along with alterations in the subchondral bone and the development of osteophytes, resulting in pain and physical disability.^34–36^ OA manifests itself in a variety of ways, including joint pain, stiffness, and restricted movement.^37^ Despite the slow progression of this disease, it can eventually cause joint failure and disability.^10^ Women between the ages of 50 and 60 years are 3.5 times more susceptible to developing hand OA than men in the same age group.^38^ Moreover, women have a 40% and 10% higher chance of developing knee OA and hip OA than men, respectively.^39,40^ In addition, initial studies suggest that women may experience more severe OA pain than men,^41^ but further research is needed.

### Estrogen

Estrogen refers to a group of steroid hormones secreted from the ovaries that play important roles in numerous tissues throughout the human body.^42^ Estrogen has significant regulatory effects not only on the development, differentiation, and function of the female reproductive system but also on the metabolism of the musculoskeletal system.^43–45^ To date, four types of estrogens have been identified in humans, namely, estrone (E1), estradiol (E2, or 17β-E2), estriol (E3) and estetrol (E4).^46,47^ E1, E2 and E3 are the most common estrogens, with E2 being the most potent and E4 only being detectable during pregnancy, as it is produced by the fetal liver.^47^ Estrogen plays a critical role in bone health in both sexes. Estrogen promotes the activity of osteoblasts, which are cells that synthesize and secrete bone matrix and participate in the mineralization of bone.^48^ This function suggests that estrogen may help slow the breakdown of bones and encourage bone growth^48^ and that reductions in estrogen over time could compromise bone health.^49^

The lipophilic nature of estrogen means that it can penetrate cell membranes and bind to estrogen receptors (ERs) with high efficiency. These receptors include ER-alpha (ERα), ER-beta (ERβ) and the membrane-bound G-protein-coupled and seven-transmembrane receptor 30 (GPR30, also named G-protein coupled ER1).^50^ To date, research has identified the presence of ERs in various joint tissues,^51^ including ERα and ERβ in human IVD cells. The ERβ gene is expressed in the annulus fibrosis of IVDs, and both ERα and ERβ are present in the nucleus pulposus and cartilage endplate.^52^ Downregulation of ERα and ERβ levels has been observed with IVD degeneration.^52,53^ GPR30 is expressed in the nucleus pulposus of IVDs and plays an important role in E2-mediated nucleus pulposus cell proliferation and survival.^54^ However, clarification of which ER subtypes are most important in IVD degeneration requires further investigation.

Chondrocyte prostaglandin synthesis can be increased by estrogen treatment, while proteoglycan synthesis can be reduced.^55^ ERs have been identified in many joint components, including synovium, bone, cartilage, and ligaments,^56,57^ suggesting that estrogen has important roles in regulating and maintaining these tissues. The influence of estrogen on cartilage metabolism has been widely studied.^58^ For example, glycosaminoglycans, as complex carbohydrates, are widely and copiously expressed on the surface of cells and within the extracellular matrix. Glycosaminoglycan synthesis is important for normal joint and cartilage function. It has been shown that glycosaminoglycan synthesis is enhanced by treating cultured rabbit chondrocytes with E2.^59^ Furthermore, cyclooxygenase-2 expression was suppressed with E2 treatment in cultured bovine articular chondrocytes, which could protect cells from oxidative damage.^60^ However, findings do not always support the positive impacts of estrogen. Studies investigating ovariectomized (OVX) rats showed that knee cartilage damage could be prevented with estrogen treatment due to effects on limiting cartilage turnover,^61–63^ whereas the cartilage structure in estrogen-deficient rabbits continued to degrade even after intraarticular injections of estrogen.^64,65^ Furthermore, higher ER expression in facet joint cartilage was thought to aggravate the severity of FJOA in postmenopausal women.^66^ These findings highlight the complex roles of estrogen in regulating the metabolic homeostasis of the IVD and facet joints and that further studies are required to disentangle the mechanisms for each estrogen and receptor subtype.

## The role of estrogen in LBP and OA pain

### Estrogen deficiency and LBP

Estrogen deficiency may facilitate IVD degeneration. In 2004, it was shown that OVX rats had lower bone mineral density and bone volume indices but a higher bone turnover rate in the lumbar spine than those of sham-operated controls.^67^ The OVX group also had higher IVD degeneration histological scores, indicative of IVD degeneration.^67^ In another study comparing OVX versus non-OVX rats, IVD degeneration induced by facetectomy appeared to be exacerbated by low estrogen levels, as the affected vertebral body (C5) was substantially weaker (confirmed by imaging) in OVX rats.^68^ In addition, IVD shrinkage, oxidative stress, and autophagy were found in rat nucleus pulposus tissue after OVX surgery.^69^ Moreover, nucleus pulposus cells in non-OVX rats remain as notochord cells, whereas in OVX rats, they differentiate into chondrocyte-like cells.^70^ Other OVX studies have also shown reductions in endplate porosity, downregulation of aggrecan, type II collagen alpha 1 (COL2α1) and Wnt/β-catenin pathways, and changed biomechanical properties of the IVD.^70^ In addition, the progression of IVD degeneration was accelerated in OVX rats through changes including osteochondral remodeling of the cartilage endplate and vertebral osteoporosis.^71^ More importantly, the calcification level of the cartilage endplate was higher in the OVX plus vehicle group than that in the sham group and OVX plus E2 group, suggesting that decreased estrogen levels aggravate the degeneration of the cartilage endplate.^72^ It is worth noting that ERα and ERβ expression was found to decrease as IVD degeneration worsened in both males and females and that ERα and ERβ expression in the nucleus pulposus of males was significantly higher than that of females.^52^ To date, however, no in vivo animal studies/human studies have investigated the impact of estrogen replacement therapy on IVD degeneration in males. This may be due to the fact that relative to females, males have very low levels of estrogen and high levels of androgens, the latter of which also has critical roles in IVD maintenance.^73,74^ Further work is needed to reveal the full impact of estrogen on IVD metabolism in both males and females.

In addition to IVD degeneration, estrogen deficiency may also promote FJOA. Researchers comparing morphologic changes in cartilage and subchondral bone of the lumbar facet joint between OVX versus non-OVX mouse models showed that OVX mice had^75^ (1) cartilage with decreased surface area, thickness, and volume; (2) surface subchondral bone damage; and (3) increased osteoclasts within their subchondral bone, which was confirmed by another study.^76^ Furthermore, lumbar facet joint arthritis was accelerated under estrogen-deficient conditions in mice, as evidenced by the severe cartilage degradation, decreased subchondral bone mass of the lumbar facet joint, increased cavities on the subchondral bone interface, and increased angiogenesis and nerve ending growth in the degenerated lumbar facet joints.^77^

Clinical evidence supports these OVX animal findings. In a clinical study, 15 OVX patients and 60 non-OVX patients with LBP were compared regarding the severity of IVD degeneration using a modified Pfirrmann grading system, a scoring tool used for IVD degeneration evaluation.^78^ Non-OVX patients had lower scores at all IVD levels than OVX patients. Unilateral OVX patients had lower scores at levels L3/4 and L5/S1 relative to those of bilateral OVX patients. These data suggest that OVX may have, at least in part, contributed to the progression of IVD degeneration. A cross-sectional study comparing estrogen levels with respect to back pain and lumbar OA among 196 postmenopausal women showed that estrogen deficiency was correlated with symptomatic lumbar OA.^79^

### Estrogen deficiency and OA

A number of studies support the role of estrogen deficiency in OA more broadly, including in the temporomandibular joint and knee joint.^80^ In OVX versus non-OVX animal studies, OVX animals had more extensive cartilage damage,^62^ erosion and subchondral bone changes,^81^ and increased surface erosion of the articular cartilage.^61,82^ Comparable alterations in cartilage structure have also been reported in other animal types, including rabbits,^83^ sheep,^84^, and guinea pigs.^85^ In 2008, a systematic review of OVX in animal OA models found that 11 of 16 studies reported significant cartilage damage in response to OVX.^86^ In a recent study, OVX mice developed severe posttraumatic osteoarthritis lesions and more extensive cartilage damage in the knee joint 8 weeks after destabilization of the medial meniscus surgery compared to non-OVX mice.^87^ These changes were supported by histological findings, including reduced safranin O staining (macroscopic observation and Mankin score), areas of defective uncalcified cartilage, and substantial decreases in both the area and thickness of the articular cartilage.^87^ In another study, the mRNA levels of early markers of osteogenic differentiation (e.g., type I collagen and Runx2) were shown to be elevated in OVX rats.^81^

Several human studies support the correlation between estrogen levels and OA.^51^ A meta-analysis found that women tended to experience more severe knee OA than men and that this was substantially amplified in women after menopause, a period characterized by marked and progressive declines in estrogen.^88^ Together with animal findings, these data highlight the potential pathological role of low estrogen levels in accelerating OA and OA pain.

### Estrogen supplementation alleviates IVD degeneration and FJOA

As shown in Table 1, the potential for estrogen to reduce IVD degeneration and FJOA progression to relieve LBP of different etiologies has been demonstrated in many animal model studies.

**Table 1:** Animal studies for the role of estrogen (17β-E2) in the relief of LBP

Rodents	Gender	Age	Treatment (*n* = 8–20)	Dose	Outcomes	Ref.
Mice (C57Bl/6j)	Female	12 weeks	Sham OVX OVX + Veh OVX + E	10 μg·kg^−1^ 5 times per week for 8 weeks, s.c.	LFJ subchondral bone turnover was efficiently suppressed; cartilage degradation was markedly inhibited; blood vessel and nerve ending growth in degenerated LFJ increased	^77^
Rats (SD)	Female	3 months	Intact + Veh OVX, OVX + Veh OVX + E OVX + P OVX + COM	4.8 μg·d^−1^ for 2 weeks s.c.	Bone loss by inhibiting the decrease in trabecular number was antagonized; trabecular thickness increased; osteoclast number and inhibited label resorption, and bone turnover reduced	^103^
Rats (SD)	Female	3 months	Sham, OVX, FR FR-OVX, ERT	10 mg·kg^−1^ 3 times/week s.c.	Vertebral bony properties were restored and IVD height loss was attenuated	^68^
Rats (SD)	Female	6 weeks	Sham, OVX + Veh OVX + E	10 μg·kg^−1^ per day for 12 weeks	Redox balance was restored; and autophagy level to prevent the IVD degeneration reduced	^69^
Rats (SD)	Female	3 months	Sham, OVX OVX + E OVX + PTH	25 μg·kg^−1^ 5 times/week for 12 weeks s.c.	The notochord cell number partly recovered; the smoothness and porosity of endplate largely recovered; biomechanical properties of IVD partly but significantly improved	^70^
Rats (SD)	Female	3 months	Sham, OVX OVX + E OVX + E + ICI	25 μg·kg^−1^ per day for 28 d	IVD height loss largely recovered; apoptosis of IVD cells were inhibited	^89^
Rats (SD)	Female	N/A	Sham OVX + Veh OVX + E	10 μg·kg^−1^ 5 d/week for 8 weeks s.c.	The cartilage endplate from calcification was protected	^72^

*17β-E2* 17β-estradiol, *BL* Baseline control group, *COM* the combination of estradiol and progesterone, *E* Estrogen, *ERT* Estrogen replacement therapy, *FR* Bilateral facet joints resection of C4-6, *LEVO* Levormeloxifene, a selective estrogen-receptor modulator, *LFJ* lumbar facet joint, *N/A* Not applicable, *OVX* Ovariectomy, *P* Progesterone, *PTH* Parathyroid hormone, *s.c.* subcutaneous, *sCT* Salmon calcitonin, *SD* Sprague Dawley, *Veh* Vehicle

The reduced density and connection of the cancellous bone of the C5 vertebral body in OVX rats with IVD degeneration were reversed after estrogen supplementation in rats with IVD degeneration.^68^ This finding may be explained by estrogen’s capacity to reduce oxidative stress and autophagy in the nucleus pulposus tissues, which is in part caused by estrogen deficiency.^69^ Thus, estrogen supplementation may be a possible therapeutic option for females with IVD degeneration. In another study, IVD shrinkage in a rat model induced by OVX was prevented by E2 treatment.^89^ It was found that the abnormal chondrocyte-like nucleus pulposus cells; reduced endplate porosity; expression of aggrecan, COL2α1, and Wnt/β-catenin pathway members; and altered biomechanical properties of IVDs caused by OVX surgery all improved after estrogen supplementation.^70^ A recent study showed that serum levels of E2 and IVD height in OVX rats increased to a similar level in the sham group after subcutaneous administration of E2 (10 μg·kg^−1^ per day).^90^ The reduced nucleus pulposus cells and ECM (including aggrecan and collagen) and ossified endplates were also reversed by E2 treatment.^90^ In another study, E2 (25 μg·kg^−1^) treatment reversed IVD cell apoptosis in OVX rats, and this E2 effect was abolished by treatment with ICI182780, an antagonist of the estrogen receptor.^89^ Moreover, targeted estrogen treatment has anti-inflammatory and antihyperalgesic effects on the nucleus pulposus tissue of IVDs in OVX mice with IVD degeneration.^91^

Estrogen replacement therapy is thought to mitigate and even prevent lumbar facet joint arthritis.^77^ Following E2 supplementation in OVX rats, arthritic pathological processes at the lumbar facet joint were suppressed, and cartilage degradation was inhibited. This result suggests that estrogen has an important role in maintaining the structural integrity of joints and that estrogen supplementation could protect against degeneration and even, in part, restore the integrity of the lumbar facet joint.^77^

The potential protective effects of estrogen on IVD degeneration in people with LBP are supported by data from clinical trials (Table 2). One study showed that menopausal women had substantially lower IVD height than premenopausal women and menopausal women receiving estrogen replacement therapy.^92^ In another study, postmenopausal women who were receiving E2 progesterone therapy, compared to those who were not, had a lower rate of lateral rotatory olisthesis, which is a potential trigger for degenerative scoliosis and LBP.^93,94^ Baron et al. showed that postmenopausal women presenting with osteoporotic vertebral fractures who were receiving estrogen-progestin treatment had significantly higher IVD heights relative to those who were not receiving treatment.^95^ Another cross-sectional study conducted in Korea examined the relationship between spinal OA prevalence and the use of hormone replacement therapy.^96^ Clearly, postmenopausal women undergoing hormone replacement therapy treatment had a lower prevalence of spinal OA. Moreover, women who had received treatment for more than 1 year were at an even lower risk than those who had received treatment for less time.^96^ Unfortunately, the authors did not provide exact details about the hormone therapies used (e.g., type or dose).^96^ These results suggest that estrogen therapy may be useful for treating LBP by limiting and even preventing the progression of IVD degeneration.

**Table 2:** Human trials for the role of estrogen in the relief of LBP

Patients involved	Gender	Age (years)	Physical conditions	Treatment		Estrogen information	Outcomes	Ref.
100	Female	54.7 (±8.1)	Menopausal	Untreated (*n* = 33)		0.625 mg CEE or different formulations of 2 mg E2 and progestin or transdermal estrogen or E2 implants	Estrogen-replete women maintained higher IVD compared to untreated post-menopausal women	^92^
		53.2 (±7.5)		ERT-treated (*n* = 44)				
		49.7 (±4.0)	Pre-menopausal	Untreated (*n* = 23)				
203	Female	48.9 (±5.1)	Pre-menopausal without fracture	Untreated (*n* = 41)		0.625 mg CEE or different formulations of 2 mg E2 and progestin or transdermal estrogen or E2 implants for a mean duration of 3.5 (±1.5) years	The hormone-treated and the premenopausal women had the highest disc heights	^95^
		57.8 (±8.9)	Menopausal without fracture	Untreated (*n* = 77)				
		52.8 (±7.4)		HRT-treated (*n* = 47)				
		70.2 (±6.3)	With osteoporotic vertebral fracture	Untreated (*n* = 38)				
146	Female	66.7 (±5.9)	Postmenopausal	Untreated or HRT-treated < 1 year (*n* = 71)		0.75–1.5 mg·d^−1^ transdermal 17β-E2 and 100 mg·d^−1^ progesterone for a mean duration of 8.7 (±6.1) years	Lateral rotatory spondylolisthesis was significantly lower in women who received HRT	^94^
		65.0 (±5.4)		HRT-treated > 1 year (*n* = 75)				
4265	Female	64.3 (±0.2)	Menopausal	HRT-treated (*n* = 588)		N/A	The HRT group demonstrated a lower prevalence of spinal osteoarthritis	^96^
				Untreated (*n* = 3 677)				
1103	Female	55–56	Postmenopausal	Untreated (*n* = 717)		N/A	Women receiving HRT had significant higher prevalence of back pain than nonusers	^188^
48	Female	43.6 (±2.4)	Premenopausal with a BMI of 21 or less and a lumbar spine BMD (L2-L4) of 1.1 ± 1 g·cm^−3^ or less	HRT-treated (*n* = 24)		2 mg E2 and 1 mg noretisteron acetate	Women receiving HRT had statistically significant decrease in nighttime back pain and the total Oswestry disability scores	^215^
		44.3 (±2.9)		Placebo-treated (*n* = 24)				

*BMD* Bone Mineral Density, *BMI* Body mass index, *CEE* conjugated equine estrogen, *E2* Estradiol, *ERT* Estrogen replacement therapy, *HRT* Hormone replacement therapy, *LBP* Low back pain, *N/A* Not applicable, *OVX* Ovariectomy

### Estrogen supplementation alleviates OA

Aside from the spine, OA occurs in many other joints, including the temporomandibular and knee joints (Tables 3 and 4).^97^ Estrogen has been shown to have important roles in the health and remodeling of joints. For example, OVX resulted in increased thickness of articular soft tissues and decreased bone volume in rats with temporomandibular joint OA.^98^ In other OVX studies involving the temporomandibular joint, E2/progesterone treatment has been shown to improve the articular disc and condylar cartilaginous layers,^99^ reduce joint inflammation,^100^ and alleviate joint nociception.^101^

**Table 3:** Animal studies for the role of estrogen in the relief of osteoarthritis pain

Joint Position	Animals	Gender	Age	Treatment (*n* = 6–15)	Estrogen	Dose	Outcomes	Ref.
TMJ	Rats (W/ SD)	Male & female	45 d	OVX + Veh OVX + E OVX + P OVX + COM	E2	50 μg·kg^−1^ per day for a week	E2 significantly reduced formalin-induced and glutamate-induced TMJ nociception in OVX female rats	^101^
	Rats (W)	Male & female	45 d	Sham proestrus Sham diestrus OVX OVX + Veh OVX + E OVX + P OVX + ICI	E2	50 μg·kg^−1^ per day for a week s.c.	E2 significantly decreased formalin-induced plasma extravasation and neutrophil migration.	^100^
	Rats (W)	Female	4 weeks	Sham OVX OVX + E	17β-E2	0.5 mg·kg^−1^ per day for one or two weeks i.m.	Replacement with 17β-E2 restored most of the histomorphometric parameters of the articular soft tissue of TMJ	^98^
	Rats (SD)	Female	3-4 months	Control OVX OVX + E	E2	0.5 mg·kg^−1^ per day for 4 weeks i.m.	A slight improvement in the cartilaginous layer thickness and proliferation of chondroid cells after ERT.	^99^
Knee joint	Rats (SD)	Female	7 months	Sham, OVX OVX + E OVX + P OVX + COM	E2	5.3 μg·kg^−1^ s.c. implanted	Increased bone formation and decreased bone resorption i.e., a low overall bone turnover status.	^216^
	Cynomolgus macaques	Female	9 years	OVX OVX + E OVX + SPE	CEE	0.625 mg·d^−1^ for 36 months Oral	The factor representing subchondral bone was significantly higher, but the number of osteophytes was lower in the ERT group. Cartilage lesions of OA were significantly less severe in the animals given ERT group.	^102^
	Rats (SD)	Female	5 months	Sham OVX + E OVX + E OVX + Low dose SERM OVX + High dose SERM	EE2	0.1 mg·kg^−1^ per day for 9 weeks Oral	E2 inhibited the ovariectomy-induced acceleration of cartilage and bone turnover and significantly suppressed cartilage degradation and erosion seen in vehicle-treated OVX rats.	^82^
	Rats (SD)	Female	6 months	Sham OVX OVX + Early E OVX + Delayed E	E2	0.25 mg pellet for 9 weeks, s.c. implanted	E2 countered the acceleration of type II collagen degradation and related structural alterations	^62^

*17β-E2* 17β-estradiol, *CEE* Conjugated equine estrogens, *COM* The combination of estradiol and progesterone, *E* Estrogen, *E2* estradiol, *EE2* 17α-ethinyl-estradiol, *ERT* estrogen replacement therapy, *F* Formalin-induced TMJ nociception, *G* Glutamate-induced TMJ nociception, *ICI* Estrogen receptor antagonist ICI 182780, *i.m.* Intramuscular injection, *LEVO* Levormeloxifene, *OVX* Ovariectomy, *P* Progesterone, *s.c.* subcutaneous, *SERM* Selective estrogen receptor modulator, *SD* Sprague-Dawley rats, *Sham* Sham surgery, *SPE* Soy phytoestrogen, *TMJ* Temporomandibular joint, *Veh* Vehicle, *W* Wistar rats

**Table 4:** Human trials for the role of estrogen in the relief of osteoarthritis pain

Patients involved	Gender	Age (years)	Physical conditions	Treatment	Estrogen dose	Joint Position	Outcomes	Ref.
10739	Female	50–79	Postmenopausal with a history of hysterectomy	E-treated (*n* = 5 310)	CEE 0.625 mg·d^−1^	N/A	After 1-year, joint pain frequency & severity were significantly lower in the estrogen-alone group than the placebo group	^104^
				Placebo-controlled (*n* = 5 427)				
4766	Female	61.63 (±6.14)	Postmenopausal	MHT-treated (*n* = 441)	N/A	Knee	The prevalence of knee OA was lower in participants with MHT than control group	^105^
		65.38 (±9.21)		Untreated (*n* = 4 325)				
1003	Female	53.5 (±4.53)	Postmenopausal or had a total hysterectomy, or hysterectomy alone and were aged over 55	Current HRT-treated (*n* = 72)	N/A	Knee & hand	An inverse association of current HRT use and radiological OA of the knee is suggestive of a protective effect. The effect was weaker in the hand joints	^107^
		53.9 (±5.29)		Ever HRT-treated (*n* = 129)				
		54.3 (±6.13)		Untreated (*n* = 874)				
26321	Female	50–79	Had hysterectomy but without a history of arthroplasty.	E-treated (*n* = 5 076)	CEE 0.625 mg·d^−1^ for an average of 7.1 years	Hip & knee	Women receiving HRT had significantly lower rates of hip arthroplasty, but not significant for knee arthroplasty	^108^
				Placebo-treated (*n* = 5 196)				
			Had no history of hysterectomy nor arthroplasty	E plus progestin-treated (*n* = 8 240)				
				Placebo-treated (*n* = 7 809)				
81	Female	58.0 (±6.1)	Postmenopausal	E-treated (*n* = 42)	N/A	Knee	Long-term using ERT had more knee cartilage than controls	^106^
		56.0 (±6.4)		Untreated (*n* = 39)				

*CEE* Conjugated equine estrogens, *E* Estrogen, *ERT* Estrogen replacement therapy, *HRT* Hormone replacement therapy, *OA* osteoarthritis, *MHT* menopausal hormone therapy, *N/A* Not applicable

OVX monkeys treated with estrogen replacement had less severe OA-related cartilage lesions within the knee joints.^102^ Similarly, knee cartilage turnover and surface erosion induced by estrogen deficiency were alleviated by estrogen supplementation in rats.^82^ Moreover, both estrogen and levormeloxifene, a selective estrogen-receptor modulator that inhibits OVX-induced changes, were found to prevent cartilage erosion, and estrogen independently prevented knee cartilage turnover in female OVX rats.^61,62^ Svetlana et al. demonstrated that OVX surgery led to reductions in serum levels of E2 and C-telopeptide of type II collagen (CTX-II) in rats but that early estrogen therapy could prevent knee cartilage turnover.^62^ Similarly, tibia bone turnover and the resultant cancellous osteopenia were reduced by E2 treatment in OVX rats.^103^

Clinical studies have shown that estrogen supplementation produces modest but sustained decreases in joint pain frequency in postmenopausal women. Indeed, joint pain prevalence and severity in postmenopausal patients were lower in those receiving estrogen compared to those receiving placebo, calcium, and vitamin D supplementation.^104^ Similar findings have been found in other large cross-sectional studies (e.g.,^105^). Furthermore, knee cartilage volume in postmenopausal women was found to be higher in estrogen therapy users than in nonusers.^106^ Consistent with these findings, the use of estrogen or estrogen-related (i.e., Prempak-C or Premarin 0.625) therapeutic agents has been associated with a lower prevalence/incidence of knee and hand OA,^107^ improved knee and hip joint health, and a lower arthroplasty rate.^108^

## Molecular mechanisms by which estrogen alleviates IVD degeneration, FJOA and OA

**Fig. 1:**
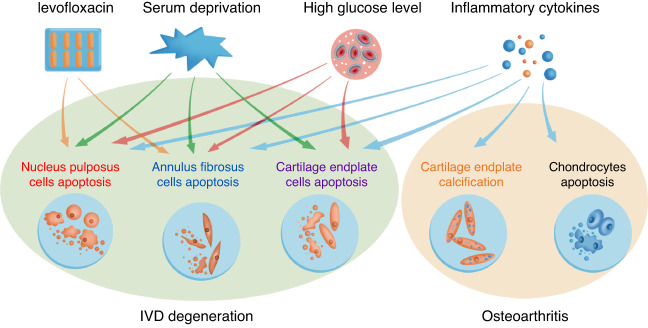
Summary of the possible estrogen-related causes mentioned in this review for intervertebral disc (IVD) degeneration and osteoarthritis

**Fig. 2:**
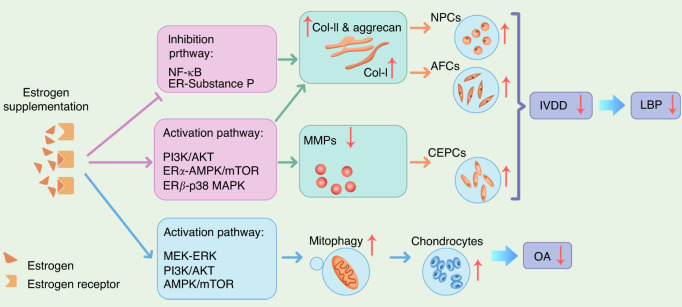
Summary of the potential mechanism by which estrogen alleviates low back pain (LBP) and osteoarthritis (OA). Both inhibition and activation pathways are involved in cell proliferation and viability. Ultimately, LBP may be alleviated by slowing intervertebral disc degeneration (IVDD). The cells involved are nucleus pulposus cells (NPCs), annulus fibrosus cells (AFCs), and cartilage endplate cells (CEPCs). OA may be alleviated by protecting and promoting the proliferation of chondrocytes through the upregulation of mitophagy. AMPK 5’ adenosine monophosphate-activated protein kinase, Col collagen, ER estrogen receptor, MAPK mitogen-activated protein kinase, mTOR mammalian target of rapamycin, NF-κB nuclear factor kappa-B, PI3K/AKT phosphatidylinositol 3-kinase/protein kinase B

Despite considerable and growing interest in the topic, the specific mechanisms by which estrogen mitigates LBP and OA remain largely unknown. The role of estrogen in IVD degeneration has been studied the most.^17,18,109–112^ There are multiple pathways by which estrogen could impact IVD degeneration, including the nuclear factor kappa-B (NF-κB) signaling pathway, phosphatidylinositol 3-kinase/protein kinase B (PI3K/AKT) pathway, ERβ-p38 mitogen-activated protein kinase (MAPK) signaling pathway, and ER–substance P signaling pathway. Pathways that are most likely involved in OA are the PI3K/AKT pathway, ERα-mitogen-activated protein kinase (MEK)-extracellular signal-regulated kinase (ERK) signaling pathway, and the AMP-activated protein kinase (AMPK)-mammalian target of rapamycin (mTOR) signaling pathway (Figs. 1 and 2).

### Molecular mechanisms underlying the role of estrogen in LBP

#### IVD degeneration-related LBP

##### Pathogenesis of IVD degeneration

The pathogenesis of IVD degeneration is complex and involves multiple factors, including spinal instability, disorganization of the concentric lamellae in the annulus fibrosus, inflammation, matrix degradation, and loss of proteoglycan content in the nucleus pulposus.^113–115^ Within the IVD, the nucleus pulposus, which consists of nucleus pulposus cells and ECM, has been robustly investigated.^116,117^ Two crucial cellular processes associated with IVD degeneration are aberrant apoptosis and accelerated aging of nucleus pulposus cells. Thus, these processes within nucleus pulposus cells have become an attractive target to prevent and treat IVD degeneration.^118,119^ Aggrecan and COL2α1 secreted from nucleus pulposus cells are the primary components that maintain the ECM, and their catabolic metabolism is mediated by the family of matrix metalloproteinases (MMPs) and influences the pathological processes of IVD degeneration.^120–124^

High levels of cytokines are synthesized by resident nucleus pulposus cells during IVD degeneration, including tumor necrosis factor α (TNFα) and interleukin-1β (IL-1β).^125^ TNFα is a proinflammatory cytokine that influences angiogenesis and nerve ingrowth of the nucleus pulposus.^126^ IL-1β is another inflammatory cytokine that contributes to the regulation and progression of IVD cell apoptosis.^127^ Thus, TNF-α and IL-1β have been widely used to induce IVD cell apoptosis in studies.^128^ As IVD degeneration progresses, other mediators, such as MMP-3 and MMP-13, play particular roles in degrading specific components of the ECM, exacerbating and worsening the condition.^129–131^

##### Effects of estrogen on the nucleus pulposus during IVD degeneration

In general, E2 prevents ECM degradation by upregulating COL2α1 and aggrecan expression while downregulating MMP-3 and MMP-13 expression in nucleus pulposus cells by regulating redox balance and autophagy.^132^ Nucleus pulposus cell apoptosis, induced by levofloxacin, has been shown to be reduced in rats treated with E2, which upregulates integrin α2β1, a subtype of the β1 integrin family that can specifically bind to COL2α1.^133,134^ The effect of 17β-E2 on IL-1β-induced rat nucleus pulposus cell apoptosis has been evaluated extensively in vitro and in vivo. E2 resisted nucleus pulposus cell apoptosis by downregulating MMP-3 and MMP-13 expression and upregulating COL2α1 expression.^90,132,134,135^ Several lines of evidence suggest that the specific mechanism is related to cellular redox balance.^136^ First, estrogen deficiency is considered to contribute to the oxidative stress and autophagy that occur in the nucleus pulposus of OVX rats.^69^ Second, autophagy is thought to protect against oxidative stress.^136^ Third, E2 restores the redox balance and reduces autophagy levels.^69^ Fourth, the interaction between 17β-E2 and ERβ was found to attenuate apoptosis (induced by high-glucose conditions) and increase matrix biosynthesis in nucleus pulposus cells in rats by inhibiting oxidative damage.^137^

***NF-κB signaling pathway***: The NF-κB family of transcription factors plays critical roles in cell apoptosis and the cellular inflammatory response.^138^ Nucleus pulposus cell apoptosis can be alleviated by inhibiting the NF-κB signaling pathway through ER-mediated genetic effects.^90^ Reactive oxygen species (ROS) generation and NF-κB activity in TNF-α-treated nucleus pulposus cells are also inhibited by E2 treatment.^138^ In this case, TNF-α induced premature senescence of nucleus pulposus cells, but these effects were reversed by E2 treatment through interference with the ROS/NF-κB pathway.^138^

***PI3K/AKT signaling pathway***: The PI3K/AKT signaling pathway has important roles in modulating cell function and multiple apoptosis-related proteins, including caspase-3, glycogen synthase kinase-3β (GSK-3β), NF-κB, and mTOR.^139–142^ AKT is a serine/threonine protein kinase that can inhibit cell apoptosis via a phosphorylation mechanism dependent on PI3K.^143^ Increasing evidence points to controlling the PI3K/AKT pathway as a potential therapeutic avenue to prevent and alleviate IVD degeneration.^144^

**(i) PI3K/AKT/mTOR/caspase-3 signaling pathway**

**Fig. 3:**
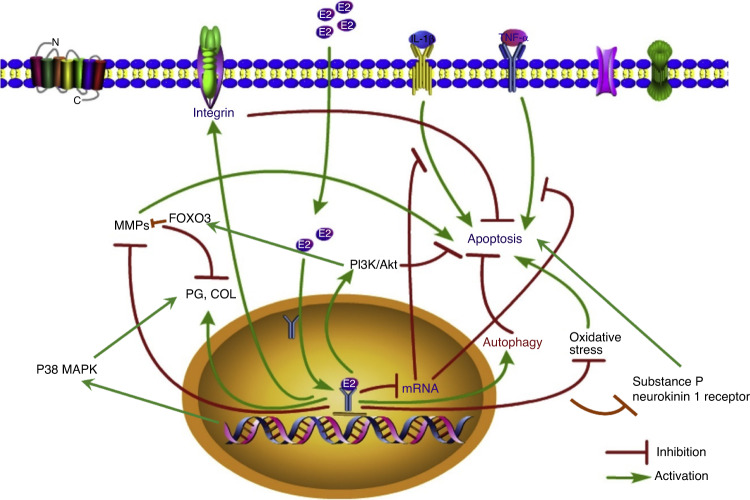
The molecular mechanism underlying the antiapoptotic effect of estrogen on intervertebral disc (IVD) cells. E2 estrogen, PG proteoglycan, COL collagen, MMP matrix metalloproteinase, IL-1β interleukin-1β, TNF-α tumor necrosis factor-α, FOXO3 Forkhead box O-3, P38 MARK p38 mitogen-activated protein kinase. Modified with permission from^109^. Copyright 2020 Elsevier

It has been demonstrated that apoptosis in human nucleus pulposus cells induced by TNF-α increases caspase-3 protein expression and decreases poly-ADP-ribose polymerase (PARP) expression levels and is alleviated by E2 in a concentration-dependent manner.^145^ This notion was supported in another study in which E2 was shown to alleviate apoptosis in the same cells (induced by TNF) through upregulation of PARP and downregulation of caspase-3 in the PI3K/AKT pathway.^146^ Our previous work revealed that E2 treatment effectively abolished the negative effects (including the upregulated COL2α1 and aggrecan and downregulated MMP-3 and MMP-13 expression) caused by IL-1β in rat nucleus pulposus cells by activating the PI3K/AKT/caspase-3 signaling pathways.^132^ Furthermore, our group has previously explored the downstream signaling pathways of PI3K/Akt and established the pathways by which estrogen protects against apoptosis. By comparing the effects of different antagonists on IL‑1β‑induced apoptosis, including ICI182780 (an ER antagonist), SC75741 (an NF-κB inhibitor), SB216763 (a GSK-3β inhibitor) and rapamycin (an mTOR inhibitor), we revealed that the anti-apoptosis effects of E2 on rat nucleus pulposus cells were reduced by ICI182780 and rapamycin.^147^ These changes were accompanied by decreases in activated caspase-3 expression and increases in mTOR expression.^147^ These results highlight the mTOR/caspase‑3 pathway as a potential avenue by which E2 protects nucleus pulposus cells against apoptosis (Fig. 3).^109,147^

**(ii) PI3K/AKT/FOXO3 signaling pathway**

Forkhead box O-3 (FOXO3) is a transcription factor, and its deficiency can accelerate IVD degeneration.^148^ It was suggested that matrix loss in human nucleus pulposus cells could be reduced by E2 via the PI3K/AKT/FOXO3 signaling pathway by decreasing MMP-3 levels.^135^ Specifically, the phosphorylation of FOXO3, caused by E2 induction of the PI3K/AKT pathway, could reduce the activity of the MMP-3 promoter and lead to increased collagen II and aggrecan expression. In addition, both FOXO3 and MMP-3 expression can be increased by the PI3K inhibitor LY294002.^135^

***ERβ-p38 MAPK signaling pathway***: The p38 MAPK signaling pathway is important in cell viability and matrix synthesis within the disc nucleus pulposus region, which can be activated by E2.^149^ The activity of the p38 MAPK pathway in nucleus pulposus cells is increased by E2 and decreased when ERβ function is blocked.^150^ Together, these findings indicate that E2 increases matrix synthesis within nucleus pulposus cells by activating the ERβ-p38 MAPK pathway and that blocking this pathway might mitigate the positive effects of E2.^150^

***The ER–substance P signaling pathway***: Substance P is a neurotransmitter and a modulator of pain perception that acts by altering cellular signaling pathways.^151–154^ The relationship between ERs and substance P in nucleus pulposus tissues has been studied in postmenopausal females who underwent posterior lumbar interbody fusion and/or disc excision.^53^ Findings confirmed that substance P colocalizes with ERα and ERβ both in the cytoplasm and nucleus of the nucleus pulposus cells and that high levels of substance P correlated with low levels of ERα and ERβ expression. That the binding of substance P with neurokinin 1 receptor (NK1R) leads to activation of NF-κB and enhanced production of inflammatory cytokines^155–157^ and that both are reduced in the nucleus pulposus cells of OVX rats in response to estrogen replacement therapy^91^ suggests that estrogen alleviates nucleus pulposus cell apoptosis by downregulating substance P.^91^

##### Effects of estrogen on the annulus fibrosus and cartilage endplate during IVD degeneration

Very few studies have investigated the potential protective effects and underlying mechanisms of E2 treatment on the annulus fibrosus and cartilage endplate. One study reported that IL-1β-induced apoptosis of annulus fibrosus cells is inhibited by 17β-E2 in a dose-dependent manner, which is abolished by an estrogen receptor antagonist.^158^ In another study, E2 was thought to alleviate rat annulus fibrosus cell apoptosis induced by IL-1β in a time-dependent manner by depressing caspase-3 activity and increasing α1 integrin-mediated adhesion to type I collagen.^159^ Moreover, transforming growth factor β1 (TGF1) could suppress apoptosis of annulus fibrosus cells by inhibiting excessive autophagy and modulating the PI3K/AKT/mTOR and ERK1/2 signaling pathways.^160^ Whether estrogen similarly affects the PI3K pathway and inhibits annulus fibrosus cell apoptosis requires further investigation.

Other causes of apoptosis of annulus fibrosus cells include levofloxacin treatment and a high-glucose environment. Levofloxacin is thought to induce these effects, in part, by decreasing the binding of annulus fibrosus cells to type I collagen and upregulating caspase-3, MMP-2, MMP-3, and MMP-13 expression.^161,162^ High-glucose environments may accelerate annulus fibrosus cell apoptosis by enhancing endoplasmic reticulum stress.^163^ Whether estrogen protects against annulus fibrosus cell apoptosis evoked by levofloxacin or high-glucose conditions has not been explored.

The cartilage endplate plays a critical role in the metabolic exchange of waste and nutrients in the IVD and thus directly influences the metabolism, proliferation, growth, and apoptosis of nucleus pulposus cells.^164,165^ Calcification of the cartilage endplate could substantially impair these processes and contribute to the development and maintenance of IVD degeneration.^166^ The potential for estrogen (or lack thereof) to impact the cartilage endplate has been investigated both in vivo and in vitro. Reduced estrogen levels have been shown to aggravate the cartilage endplate by inducing its calcification, which is mitigated by E2 treatment in a dose-dependent manner.^72,167^ E2 treatment has been shown to enhance aggrecan and COL2α1 expression and the proliferation of cartilage endplate cells and can inhibit cell apoptosis within the cartilage endplate.^167^ Furthermore, the effects of E2 could be attenuated by microRNA (miR)-221 or knockdown of ERα, a target of miR-221, which suggests that miR-221 could be involved in the protective effects of E2 on cartilage endplate degeneration.^167^ Moreover, apoptosis of cells and calcification of the cartilage endplate occurs under oxidative stress conditions (induced by H_2_O_2_) via the ROS/p38/ERK/p65 pathway.^168^ Other conditions/scenarios, such as high-glucose environments and serum deprivation, also promote apoptosis of cartilage endplate cells via mitochondrial mechanisms.^169^ Serum deprivation increased apoptosis of cartilage endplate cells,^170^ and this process has been shown to be induced by the mitochondrial intrinsic pathway.^171^ However, research on the role of estrogen in these processes warrants further exploration.

#### FJOA-related LBP

FJOA is considered a consequence of spinal degeneration and another potential source of LBP. The specific molecular mechanism of estrogen on FJOA is rarely reported, but we can draw inspiration from some other studies.

Chen et al. reported that increased angiogenesis and neurogenesis were observed in OVX-induced LFJ degeneration.^77^ In contrast to nondegenerated knee joints, rheumatoid arthritis and OA have higher rates of osteochondral angiogenesis, which is controlled by a proangiogenesis microenvironment in the subchondral bone marrow.^172^ Fibrovascular replacement of marrow tissue is a process in which new blood vessels form in the subchondral bone, which is the layer of bone just beneath the articular cartilage in joints. This process is associated with the production of various angiogenic cytokines, such as IL-1α, IL-8, IL-10, vascular endothelial growth factor, and platelet-derived growth factor.^173,174^ Neovascularization and innervation are often closely associated, particularly in the context of joint degeneration and arthritis.^175^ The colocalization of nerve growth factor with an increased density of nerve fibers in joint degeneration has been identified as a contributing factor to the sensation of arthritis pain by sensitizing the peripheral nerve endings.^176^ Therefore, it is supposed that estrogen plays important roles in angiogenesis and neurogenesis against FJOA via angiogenic cytokines, which needs further verification. In addition, cartilage is one of the major elements in joint structure. ERα is expressed in different components of the LFJ, including chondrocytes and subchondral bone marrow cells.

### Molecular mechanisms underlying the role of estrogen in OA

The accelerated cartilage degeneration and cell apoptosis that occur in people with OA are, at least in part, due to weakened mitophagy in chondrocytes in the articular cartilage.^177^ Chondrocyte apoptosis and cartilage degeneration are accelerated within the articular cartilage when mitophagy function is impaired in resident chondrocytes.^177^ As an important inhibitor of autophagy, mTOR is a crucial signaling molecule downstream of AMPK. In addition, the levels of mTOR could be inhibited by an increase in its phosphorylation, promoting autophagy.^178^ AMPK is a key molecule in chondrocyte metabolism and can regulate metabolism via mediators including mTOR and Sirtuin-1 (SIRT1), an NAD^+^-dependent type III histone deacetylase.^179^ SIRT1 plays an important role in protecting chondrocytes, and the apoptosis of chondrocytes could be protected by SIRT1-mediated autophagy.^180–182^ Patients with OA have elevated synovial fluid concentrations of nerve growth factor (NGF),^183^ which can be amplified by cartilage damage. NGF could upregulate FGF2 (fibroblast growth factor 2) expression in a time- and dose-dependent manner to promote endothelial cell migration and tube formation through the PI3K/Akt and ERK/MAPK pathways in human chondrocytes.^184^

***PI3K/AKT signaling pathway***: There is evidence that E2 alleviates OA chondrocyte apoptosis and promotes the proliferation of chondrocytes and the expression of AKT and P-AKT.^185^ That these effects are reduced by a P-AKT inhibitor further highlights the PI3K/AKT pathway as a potential mechanism by which E2 reduces apoptosis and enhances the proliferation of chondrocytes.^185^

***ERα-MEK-ERK signaling pathway***: In a recent study, E2 reduced NGF expression within chondrocytes in both rat knee cartilage and cartilage explants, which could be reversed by a specific ERα inhibitor (MPP). This finding suggests that estrogen downregulated NGF expression via ERα. Furthermore, the induction of NGF expression by TGF-β1 or IL-1β in rat chondrocytes could be decreased by estrogen but increased by PD98059, an inhibitor of the MEK-ERK pathway. This result suggests that the MEK-ERK signaling pathway may be mediated by the modulatory effects of E2 on NGF.^186^

***AMPK/mTOR signaling pathway***: Increased levels of mitophagy-related proteins (SIRT1 and p-AMPK) and decreased levels of p-mTOR proteins have been observed in rats following 17β-E2 treatment.^180^ Other observations in these rats posttreatment included increased mitochondrial autophagosomes and activation of the AMPK/mTOR signaling pathway. These findings provide evidence that E2 may induce mitophagy to protect chondrocytes through the SIRT1-mediated AMPK/mTOR signaling pathway.^180^

## Controversial results and risks associated with estrogen treatments

Although most evidence points to estrogen as a possible therapy to better manage and perhaps even prevent LBP and OA pain, some studies report the opposite results and cannot be neglected. For example, there is prospective evidence that postmenopausal estrogen use is associated with an increased risk of LBP and impaired back function in elderly women.^187^ Another large study provided cross-sectional evidence that 1 103 women between 55 and 56 years of age receiving hormone replacement therapy had a slightly higher prevalence of LBP than those not receiving hormone replacement therapy.^188^ Increased risk of chronic LBP has also been found for long-term use of systemic menopausal hormone therapy, especially for the replacement of estrogen.^189^ Several factors may explain these findings. First, individuals who are more attentive to their symptoms, prone to experiencing pain, and/or experiencing severe climacteric symptoms would be exposed to more opportunities to receive hormone replacement therapy.^190^ Second, sex steroids and hormone replacement therapy can alter nociceptive processing at various levels of the nervous system, from the peripheral to the spinal and rostral regions of the central nervous system.^110^ At the peripheral level, estrogen can impact the receptive field properties of primary afferents. At the central level, estrogen can interact with numerous neurochemical systems, such as the endogenous opioid system, which could impact descending pain control.^191^ Longitudinal trials that test the efficacy of estrogen-related treatments and the underlying mechanisms in clinical populations are needed to address these issues.

Similar observations have been reported in OA. Data from two of the most extensive longitudinal observational studies ever undertaken showed that estrogen treatment did not considerably decrease the radiographic severity of knee or hand OA.^107,192,193^ That estrogen might differentially impact OA depending on the stage of the condition could explain these results. For instance, early OA is characterized by accelerated bone turnover and consequent bone loss, whereas late-stage OA is characterized by decelerated bone turnover and more subchondral sclerosis.^194,195^ Thus, estrogen may positively protect against high bone turnover during early OA but prevent the required bone turnover needed to avoid pathological bone thickening during late-stage OA. Moreover, estrogen might contribute differently to specific types of OA. For instance, the role of estrogen within the carpometacarpal joint is unclear.^107^ Conversely, estrogen has been shown to mediate inflammation in the temporomandibular joint via the NF-kB pathway in a dose-dependent manner.^196^ Finally, subchondral bone remodeling can result in either an osteoporotic or sclerotic phenotype depending on the ratio between bone formation and resorption. These findings highlight the need to better understand the role of estrogen as OA develops and progresses over time for different joints to more accurately assess if, how and when estrogen interventions will and will not be helpful in preventing and/or managing OA.

In addition, it is important to consider that some diseases and conditions have been associated with estrogen. For instance, a randomized control trial of 16 608 women concluded that the overall health risks exceeded the benefits from the use of estrogen therapies in postmenopausal women.^197,198^ Some of these risks include venous thromboembolism,^199^ coronary heart disease and cancer,^200^ although these are usually reported with long-term (e.g., >5 years) therapy (e.g.,^201^). As a result, the clinical safety of estrogen replacement therapy has also faced criticism. In response, alternative substances that can mimic estrogen’s effects have gained attention.^197^ Two examples include selective estrogen receptor modulators (SERMs) and phytoestrogens.^202–204^ SERMs are compounds that interact with estrogen receptors in specific tissues, producing estrogenic effects in some areas but anti-estrogen effects in others.^202,205^ This selective action takes advantage of the beneficial effects of estrogen, such as preventing bone loss, and limits adverse effects associated with traditional estrogen therapy.^202,206^ The treatment efficacy of SERMs has been studied in various medical conditions, including breast cancer,^207^ osteoporosis,^208^ and cardiovascular disease.^209^ Raloxifene is an example of a SERM that has been approved by the FDA for the treatment of osteoporosis in postmenopausal women.^210^ It works by mimicking the positive effects of estrogen on bone density, thereby reducing the risk of fractures.^210^ Moreover, raloxifene has also shown promise as a prophylactic (preventive) treatment for painful IVD degeneration.^210^ Although raloxifene and other SERMs offer potential benefits over estrogen therapy, their use should be evaluated on an individual basis. Unlike SERMS, phytoestrogens are naturally occurring plant compounds that have estrogenic activity and are found in various foods, such as soybeans, flaxseeds, and legumes. Phytoestrogens have been investigated for their potential health benefits, including reducing menopausal symptoms,^211^ improving bone health,^212^ and providing cardiovascular protection.^213^

Another critical factor is the dosage of estrogen treatment. On the one hand, estrogen treatment at higher doses (>500 μg·kg^−1^ per day) has been shown to be more effective at reversing cartilage degeneration than treatment at lower doses (<500 μg·kg^−1^ per day).^214^ On the other hand, excess levels of estrogen can lead to autoimmune diseases and cancer, especially breast cancer.^200^ Further studies are needed to clarify the optimal threshold level of estrogen dosage and the biological mechanisms that drive these positive and/or negative changes, some of which appear to strongly depend on the level of estrogen.

## Conclusions

Many studies have reported on the role of estrogen in LBP and OA pain. Of all the estrogen subtypes, E2 is the most potent and is considered to have the most significant role in IVD degeneration, FJOA pain, and OA pain. This review draws on these studies to provide a current update on the relationship between estrogen and LBP/OA pain and the mechanisms that underlie them. Growing evidence continues to reveal critical roles for estrogen in maintaining healthy IVDs, joints and various tissues implicated in LBP and OA. This has opened the door for exploring estrogen as a potential therapeutic option to prevent and manage LBP and OA, especially during low estrogen periods as can occur after menopause. However, the pathways and mechanisms by which estrogen alleviates LBP and OA pain are not yet clear, and the potential side effects of estrogen therapy are still being revealed. Further investigation is warranted to close these knowledge gaps with a focus on exploring which estrogen treatments and at what doses are most effective and safe for different pain conditions at different stages of the condition.
